# Long-term effects of caesarean delivery on health and behavioural outcomes of the mother and child in Bangladesh

**DOI:** 10.1186/s41043-022-00326-6

**Published:** 2022-10-04

**Authors:** Mostafizur Rahman, Nuruzzaman Khan, Aminur Rahman, Mahmudul Alam, Alam Khan

**Affiliations:** 1 Department of Population Science and Human Resource Development, University of Rajshahi, Rajshahi, Bangladesh; 2 Department of Population Science, Jatiya Kabi Kazi Nazrul Islam University, Trishal Mymensingh, Bangladesh; 3 Department of Statistics, University of Rajshahi, Rajshahi, Bangladesh; 4 Department of Pharmacy, University of Rajshahi, Rajshahi, Bangladesh

**Keywords:** Caesarean delivery, Adverse health effects, Multivariate logistic regression model, Bangladesh

## Abstract

**Background:**

Medically unnecessary caesarean section (CS) is now an ongoing concern worldwide including in Bangladesh. This intensifies the occurrence of adverse maternal and child health outcomes. We investigated the associations of CS with some basic health and behavioural outcomes of the mothers and their children in Bangladesh.

**Methods:**

We conducted a community-based case–control study from May to August 2019. A total of 600 mother–child dyads were interviewed using a structured questionnaire, 300 of them had CS, and 300 had vaginal delivery (VD) in their most recent live births. The exposure variable was the mode of delivery, classified as 1 if mothers had CS and 0 if mothers had VD. The outcome variables were a group of health and behavioural problems of the mothers and their children. Multivariate or multiple logistic regression model, separately for each health and behavioural outcome, was used to determine the effect of exposure variable on outcome variable after adjusting for possible confounders.

**Results:**

The mean age and weight of mothers were 25.1 years and 53.1 kg, respectively. Likelihoods of headache, after delivery hip pain, problem of daily activities, and breastfeeding problem were reported higher among mothers who had CS in their most recent live birth than mothers who had VD. Similarly, children who were born through the CS operation were more likely to report breathing problem, frequent illness, lower food demand and lower hours of sleeping.

**Conclusion:**

The use of CS increases the risks of health and behavioural problems of the mothers and their children. Policies and programs to avoid medically unnecessary CS and increase awareness over its adverse effects are important.

## Background

Caesarean section (CS) is a surgical procedure in which a baby is delivered through an incision of the mother’s abdomen. Ideally, this is recommended when the Vaginal Delivery (VD) would put a baby and/or his/her mother at risk and would not be saved without performing the CS. The World Health Organization (WHO) in 2004 recommended an upper limit of CS use should be 15%, with a further recommendation of 1–5% CS use is enough to avoidable unnecessary morbidity and mortality of mothers and their children [1, 2]. However, the number of CS is increasing globally, starting in early 1990 [3]. Around 21% of the total current world’s births, ranging from 6% in low- and middle-income countries (LMICs) to 27% in developed region, are currently ended by CS [3]. At the regional level, the rate is higher in Eastern Asia (35%), Central America (38%), North America and Oceania (32%) [3, 4]. These rates would have been increased to around 50% by 2030 with a global prevalence of 29% CS- a recent projection found [4]. The reasons for these dramatic increases are multi-factorial and varied across countries’ socio-economic conditions. However, maternal demographic and reproductive characteristics, including higher age mothers at the time of pregnancy, higher birth order, lower inter-pregnancy interval, higher household wealth quintile and higher educational status of the mothers and their partners are found most influential factors of performing CS in Bangladesh as well as other LMICs [5–7]. Professional practice style of the health care personnel, including unethical practice to motivate mothers to undergo CS for making money, has recently been addressed as other important contributors to the rising rate of CS in LMICs [8–10]. Moreover, mothers’ homes distance from the nearest health care facility and quality and accessibility of the health care services available there are found as other important predictors of CS use in a recent study of Bangladesh [7].

There is evidence that the use of CS prevents nearly 187,000 maternal and 2.9 million neonatal deaths worldwide [4, 11]. However, along with these contributions in reducing maternal and child deaths, medically unnecessary CS can increase short- and long-term health risks of mothers and their children. The short-term risk includes infection, haemorrhage, visceral injury, placenta accrete, and placental abruption [12–14]. The long-term risk includes asthma and obesity [12]. Moreover, higher likelihoods of miscarriage, ectopic pregnancy, and stillbirths in the subsequent pregnancies are found among the mothers having CS [15–20]. Placental accrete, placental abruption, and uterine rupture are also found higher among mothers with a previous history of CS than VD [18, 21, 22]. However, the effects of CS on maternal and child physical and behavioural health problems following delivery have not been studied yet, in Bangladesh, as well as, in other LMICs. Therefore, in this study, an attempt has been made to examine the effects of CS on some basic physical and behavioural health problems of mothers and children. The findings of this study will inform the policymakers in making relevant policies and programs and will work as a basis for conducting relevant research in a larger setting in future.

## Methods

### Data collection

A community-based retrospective case–control study was conducted from May to August 2019. The study was conducted in seven communities of the Rajshahi District of Bangladesh, covering both the village and suburban area. Around 60,000 people live in our study area whereas the community health workers reported 2560 live births within three years before the survey was conducted as part of the immunization program. We used this list as the sampling frame. We first divided this list as per the delivery methods (CS vs. VD). Data were then collected from 600 mother–child dyads in total, covering an equal number of CS (*n* = 300) and VD (*n* = 300). Our included sample was three times higher than the standard sample size required for this study as determined using the standard formula for required sample size calculation [23]. The samples were selected randomly by using the list and continued up to the inclusion of 300 mother–child dyads in each of the CS and VD group. Data were collected through face-to-face interviews by using a standard questionnaire, which was first developed in the English language and later translate to Bengali (the native language of Bangladesh). Eight Bachelor of Science (B.Sc.) students collected data under the supervision of the principal investigator, the first author of this paper, who also trained properly all data collectors before starting data collection.

### Exposure variable

The main exposure variable was the mode of delivery (CS vs. VD) that mothers used in their most recent live birth. Other exposure variables considered were maternal age of birth, place of residence, wealth quintile, education status of the mothers and their partners, number of children ever born, and mothers’ body mass index. These variables are found as important determinants of CS in national-level studies of Bangladesh [7, 24] as well as other LMICs.

### Outcome variables

We considered a range of maternal and child health outcomes as outcome variables. Mothers’ health outcomes were headache (pain in the head or neck), after delivery hip pain (pain in the buttock or lower back), problem of daily activities (problems associated with daily work including cooking and walking), suffering physical problems (problems in any organ of the body including eye problem, backbone problem), and breastfeeding problem (women reported they were faced problem during breastfeeding). The type of physical health problems that women faced was also explored and the relevant variable was classified as no problem, eye problem, backbone problem. Breathing problem (problem during breath), frequent illness (measured by at least once quarterly), behavioural characteristics (classified as obstinate, restless, and quiet), food demand (5–6 times in a day for children under 2 years old and 4–5 times a day for the children above 2 years is considered as normal), sleeping hours (40% times a day is considered normal) were considered as child health outcomes. The relevant data were collected by asking the mothers about their health problems after the most recent delivery to the survey date. Child data were also collected from them by asking a separate set of questions. Precautious measures were taken to reduce the recall bias. As part of this, all respondents were asked to show the relevant health cards where available. If the health care cards were not available, respondents were asked several follow up questions to ensure respondents reported correct answers, as such, to reduce the recall bias.

### Statistical analysis

We used mean and standard deviation to describe participants’ characteristics. The Pearson’s Chi-squared (*χ*2) test was used to find the significance of the difference of maternal and child health outcomes across methods of delivery (CS vs. VD). Association between outcome variables with methods of delivery was determined after adjusting for socio-demographic characteristics of the mothers. Multivariate logistic regression was used for this purpose if the outcome variable were dichotomous (yes, no). Multiple logistic regression model was used if the outcome variables had more than two categories. All results are reported as Odds Ratio (OR) and its 95% confidence interval (95% CI). All analyses were performed using the Statistical Package for Social Science (SPSS) version 20.0.

### Ethical consideration

The Institutional Animal, Medical Ethics, Biosafety and Biosecurity Committee (IAMEBBC) of the Institute of Biological Science, University of Rajshahi, Bangladesh, reviewed and approved this study (approval number is 78/318/IAMEBBC/IBSc). Informed verbal consent was obtained from the study participants after exploring the objectives of this study. The confidentiality of the participants was ensured as well.

## Results

The mean age of the mothers was 25.1 years and the mean weight was 53.14 kg (Table 1). A majority of the respondents had primary education (mean year of education was 5.40 years) and they had at least two children (mean number of children ever born was 1.9) at the time of the survey conducted. The mean age of marriage was around 16 years.

**Table 1:** Background characteristics of the respondents

Characteristics	Subject (*N*)	Crude
*Mean(SD)*		
Maternal age, years	600	25.1 (± 5.2)
Weight(In Kg)	600	53.14 (± 7.2)
Education(years)	600	5.40 (± 2.9)
Children ever born	600	1.9 (± 1.1)
Age at first marriage	600	15.9 (± 3.1)
Number of antenatal visit	600	3.22 (± 1.3)

The self-reported health problems among the mothers and their children across modes of delivery (CS vs. VD) are presented in Tables 2 and 3, respectively. Adverse mothers and child health outcomes were found higher among the mothers who performed CS and children born by CS. These differences were to be statistically significant at less than 5% level.

**Table 2:** Mothers’ physical health problems by mode of delivery

Physical problem	Vaginal delivery mothers,% (*n* = 300)	Caesarean delivery mothers,% (*n* = 300)	*p*-value
*Headache*			
Yes	140 (46.7)	250 (83.3)	< 0.01
No	160 (53.3)	50 (16.7)	
*After delivery hip pain*			
Yes	108 (36.0)	260 (86.7)	< 0.01
No	192 (64.0)	40 (13.3)	
*Problem of daily activities*			
Yes	60 (20.0)	280 (93.3)	< 0.01
No	240 (80.0)	20 (6.2)	
*Suffering physical problem*			
Yes	120 (40.0)	280 (93.3)	< 0.01
No	180 (60.0)	20 (6.7)	
*Types of physical health problem*			
No problem	150 (50.0)	20 (6.7)	< 0.01
Eye problem	50 (16.7)	70 (23.3)	
Backbone pain	70 (23.3)	180 (60.0)	
*Breast feeding problem*			
Yes	111 (37.0)	250 (83.0)	< 0.01
No	189 (63.0)	50 (17.0)

**Table 3:** Child health problems by mode of delivery

Categories	Vaginal delivery baby,% (N = 300)	Caesarean delivery baby,% N = 300	*p*-value
*Breathing problem*			
Yes	60 (20.0)	217 (72.3)	< .01
No	240 (80.0)	83 (28.0)	
*Frequent illness*			
Yes	52 (17.3)	230 (76.7)	< .01
No	248 (82.7)	70 (23.3)	
*Behavioural characteristic*			
Obstinate	80 (26.7)	20 (6.7)	< 0.05
Restless	80 (26.7)	160 (53.4)	
Quite	140 (46.7)	120 (40.0)	
*Food demand*			
Little	44 (14.7)	240 (53.3)	< 0.01
Normal	256 (85.3)	60 (20.0)	
*Sleeping tendency*			
Few	78 (26.0)	80 (26.7)	< 0.01
Normal	222 (74.0)	220 (73.3)	

We run a series of multivariate logistic regression models to examine the effects of CS on different maternal and child health outcomes (Table 4). We found mothers who had CS than VD reported a 3.57 (95% CI, 2.34–4.62) times higher likelihood of headache and 3.26 times (95% CI, 2.80–4.24) higher likelihood of hip pain. Mothers who had CS operations in their most recent pregnancies were 2.68 times (95% CI, 1.90–3.87) more likely to report the problem in daily activities than their counterparts who had VD. The likelihood for backbone pain (OR, 3.58, 95% CI, 2.12–4.70) was found higher among mothers having CS than VD. Moreover, mothers who had CS were more likely to report the problem in breastfeeding (OR, 3.19, 95% CI, 2.90–4.20) than mothers who had VD.

**Table 4:** Odds ratio of mothers’ and children’s health problems among mothers who have performed caesarean delivery either vaginal delivery

Mothers’ health problems	Odds ratio (95% CI)	Children’s health problems	Odds ratio (95% CI)
*Headache*		*Breathing problem*	
Vaginal delivery	1	Vaginal delivery	1
Caesarean delivery	3.57 (2.34–4.62)**	Caesarean delivery	2.62 (1.90–3.67)*
*After delivery hip pain*		*Frequent illness*	
Vaginal delivery	1	Vaginal delivery	1
Caesarean delivery	3.26 (2.80–4.24)**	Caesarean delivery	5.10 (3.90–6.20)**
*Problem of daily activities*		*Behavioural Characteristics*	
Vaginal delivery	1	Obstinate	1
Caesarean delivery	2.68 (1.90–3.87)**	Restless	0.45 (0.12–0.90)**
*Suffering physical problem*		Quite	1.93 (1.01–2.89)**
Vaginal delivery	1	*Food demand*	
Caesarean delivery	4.15 (2.90–4.12)*	Vaginal delivery	1
*Types of physical health problem*		Caesarean delivery	0.45 (0.12–0.98)**
No problem		*Sleeping tendency*	
Eye Problem	1.38 (0.95–4.20)	Vaginal delivery	1
Back bon pain	3.58 (2.12–4.70)**	Caesarean delivery	0.69 (0.20–1.00)
Others	1.28 (0.90–2.20)		
*Breastfeeding problem*			
Vaginal delivery	1		
Caesarean delivery	3.19 (2.90–4.20)**		

Notes: **p < 0.05, *p < 0.01. Each model adjusted for maternal age of birth, place of residence, wealth quintile, education status of the mothers and their partners, number of children ever born, and mothers’ body mass index

The children of the mothers who had CS were reported 2.62 times (95% CI, 1.90–3.67) higher odds of breathing problem after delivery than the children of the mothers who had VD. Around 5 times (OR, 5.10; 95% CI, 3.90–6.20) higher likelihood of frequent illness was also found among children born by CS than VD. In addition, children of mothers who had CS than VD were reported a higher odd of quite behaviour (OR, 1.93, 95% CI, 1.01–2.89). The likelihoods of normal food demand and normal sleeping hour were found to be 55% (OR, 0.45, 95% CI, 0.12–0.98) and 31% (OR, 0.69, 95% CI, 0.20–1.00) lower, respectively, among children whose mothers had CS than the children whose mothers had VD.

## Discussion

Through this study, we determined the effects of CS on the health and behavioural problems of mothers and their children. We reported a higher likelihood of health and behavioural problems among mothers who had CS and children who were born through CS. As far we know this observation is first in Bangladesh, as well as in other LMICs. Policies and programs from the governmental level to prevent medical unnecessary CS are important.

The use of CS was found associated with improvement in maternal and child health outcomes up to 1985, when the rates of CS were around 10–15% [25]. However, when the rates were crossed to 15% in the earlier part of 1990, it becomes a concern, since, such increasing rates of CS were showed no evidence of further improvements in maternal and child health [25, 26]. Even such increasing rates of CS, which are mostly performed without medical necessity, are found associated with obstetric complications and associated maternal and neonatal morbidities and mortalities [27]. A 14 years follow-up study in Canada of more than 2 million women found a 3 times higher risk of mortality from obstetric complications, including blood clots and heart attacks among the mother performed CS unnecessary [28]. Similarly, a higher likelihood of respiratory distress, metabolic and immune diseases are found among children born by CS than VD [28, 29]. There is also evidence that many of these adverse outcomes take a long time to recover and sometimes it stays lifelong [4, 19, 20, 29].

We found higher adverse health and behaviour problems are more common among the mothers who had CS as well as among their children. More than 83% of the mothers who had CS reported headaches and hip pain. These adverse outcomes are associated with anaesthesia. During CS, the doctor inserts a needle in the mother spinal area for administrating the pain medication. However, insertion punctures cover the spinal cord which leads to spinal fluids seepage and causes post-surgery headache [30–32]. Mothers suffering from this problem are mostly recovered within a week after delivery [31]. However, in some cases, the problem can stay longer period and could influence women daily activities and other associated physical health problems. This study also reported their higher likelihoods among the mothers who had CS than VD, which was similar to the findings of a study conducted in Australia [33]. Using post-partum care following the CS is important to overcome these complications. However, this is a further challenge in Bangladesh and other LMICs as post-partum care use is very low and many women consider these adverse outcomes are only for short time with no effective remedy [34].

We found CS mothers are more likely to face problems in exclusive breastfeeding—a similar finding reported in other LMICs [35–38]. The reason is lower earlier skin contact of mothers and children soon after delivery [39]. Mothers earlier return to the formal work, which is common among CS mothers as they are usually educated and belong to higher socioeconomic status, should be another predictor of lower exclusive breastfeeding following the CS [35, 39]. Importantly, lower exclusive breastfeeding is responsible for several adverse health outcomes of children, including frequent illness, lower food demand, and higher nutritional disorders that we also found in this study as similar to a previous study [35]. Researchers of a recent nationally representative study in Bangladesh concluded lowering exclusive breastfeeding is associated with increased infectious diseases among children, including diarrhoea and acute respiratory infection [40]. In addition, in this study, breathing problems, quite behavioural characteristics, and lower sleeping hours were found following the CS than VD. These adverse outcomes should be considered as consequences of lower exclusive breastfeeding that we found in this study.

This study has several strengths and some limitations. To our knowledge, this is the first study of its kind that considered CS and a range of adverse health and behavioural outcomes of mothers and their children. The associations were adjusted with the range of confounders’ factors which make this study findings more precise and should be used for policymaking in Bangladesh as well as in other LMICs. However, our relationship is correlational only rather than causal. Moreover, the data for this study were collected for some selected communities; therefore, the associations reported should not be representative to all areas of Bangladesh.  In addition, data were collected by asking respondents a series of relevant questions. Relevant health care cards were also checked. However, instead of these pre-cautious measures, recall bias is still a major issue for this study. However, any such errors is likely to be random.

## Conclusion

The use of CS increases the risk of several adverse health and behavioural outcomes of the mothers and their children. The risks of headache, after delivery hip pain, the problem of daily activities, suffering physical problems, and breastfeeding problem were found higher among mothers who had CS in their most recent pregnancies than mothers who had VD. In addition, children born by the CS reported a higher risk of breathing problems, frequent illness, and quite behavioural characteristics. Lower risk of normal food demand and lower sleeping hours were also found among children born by CS than children born by VD. Policies and programs to prevent unnecessary CS and ensure frequent health check-ups of the mothers and their children following the CS operation are important.

## Data Availability

The ethical review broad impose restriction to share this data publicly. However, data can be made available upon reasonable request to the corresponding author and approval from the ethical review broad.

## Abbreviations

CS: Caesarean section
VD: Vaginal delivery
WHO: World Health Organization
LMICs: Low- and middle-income countries
